# Lichen Planus Pigmentosus Localized to the Glans Penis: A Rare Presentation Treated With Ruxolitinib

**DOI:** 10.7759/cureus.102909

**Published:** 2026-02-03

**Authors:** Sanjidah Ira, Hannah Kopelman, William Steffes

**Affiliations:** 1 Dermatology, New York Institute of Technology College of Osteopathic Medicine, Glen Head, USA; 2 Dermatology, Kansas City University Graduate Medical Education Consortium Advanced Dermatology Cosmetic Surgery Orlando Program, Orlando, USA

**Keywords:** genital lichen planus pigmentosus, glans penis, janus kinase inhibitor, lichen planus pigmentosus, ruxolitinib

## Abstract

Lichen planus pigmentosus (LPP) is a rare variant of lichen planus characterized by macules of black or dark brown color distributed in the sun-exposed areas of the body. It is common among individuals with darker skin, with cases reported in the Middle East, Korea, Japan, and Latin America. Genital involvement is rare, with no reported cases describing isolated penile disease. Management of genital LPP is also challenging due to the sensitive nature of the skin and the associated risks of long-term topical corticosteroid use. We report a case of a 24-year-old male presenting with asymptomatic hyperpigmented lesions localized to the dorsal penile shaft, diagnosed as LPP on histopathology. The patient demonstrated minimal response to prior therapies, including topical corticosteroids and topical calcineurin inhibitors (TCIs). Given treatment resistance, topical ruxolitinib was initiated, with subtle improvement. This case highlights a rare anatomic presentation of LPP and explores the potential role of topical JAK inhibitors as a novel therapeutic option for lichen-planus-associated pigmentary disorders.

## Introduction

Lichen planus pigmentosus (LPP) is a rare variant of lichen planus, characterized by hyperpigmented macules and patches, most commonly affecting sun-exposed areas such as the face, neck, and axillae. Unlike classic lichen planus, which typically presents with pruritic purple papules and may involve the nails and scalp, LPP is primarily a pigmentary disorder with subtle surface changes, making early recognition increasingly challenging. It typically presents in adulthood, with insidious onset after age 30 [1]. It occurs in both sexes but shows a female preponderance [1]. The initial lesions are small, brown macules with diffuse borders, but they later merge to form larger pigmented areas [1]. LPP can also rarely present as macular pigmentation of the flexures in lighter-skinned individuals [2].

Histologically, LPP demonstrates an atrophic epidermis along with vacuolar degeneration of the basal cell layer [1]. A related variant of LPP is LPP inversus, which has been found to occur in non-sun-exposed intertriginous or skinfold areas of the body and predominantly occurs in Caucasians [1]. The histology is similar to that of LPP. Due to LPP having significant clinical and histological similarity with erythema dyschromicum perstans, also known as ashy dermatosis and pigmented contact dermatitis, all of which present with acquired dermal hyperpigmentation, it is a difficult diagnosis to make in routine practice [2]. Furthermore, genital involvement is exceptionally rare because LPP typically presents in photo-distributed areas, and there are currently no reports of isolated penile LPP. This makes isolated penile localization diagnostically challenging.

Therapeutic options for LPP lack robust evidence and have demonstrated variable results. These include low-potency topical steroids for short-term use and topical calcineurin inhibitors (TCIs) such as tacrolimus or pimecrolimus. Management is particularly challenging on genital skin due to the risk of corticosteroid-induced atrophy and irritation, as well as the potential psychosocial distress associated with visible genital pigmentary changes, emphasizing the need for effective steroid-sparing approaches. Furthermore, since lesion clearance is seldom complete, lasers, in combination with topical agents, can be considered [2]. Recent advances in the treatment of inflammatory skin disease highlight the Janus kinase-signal transducer and activator of transcription (JAK-STAT) pathway as an important mediator of interferon-driven cytotoxic T-cell inflammation [3]. Ruxolitinib, a topical JAK inhibitor, has shown promise in treating inflammatory and pigmentary dermatoses, including vitiligo and atopic dermatitis. However, its use in LPP remains underexplored. Here, we report the rare case of LPP on the penis, treated with topical ruxolitinib, highlighting a possible steroid-sparing therapeutic strategy in a sensitive anatomic location.

## Case presentation

A 24-year-old male presented with asymptomatic hyperpigmented lesions of the penis that began in December 2021 and were present for four months at the time of initial evaluation. The patient denied pruritus, pain, dysuria, systemic symptoms, new medications, recent infections, or exposure to new personal care products. Examination revealed gray-brown hyperpigmented annular plaques without erosion, ulceration, or Wickham striae on the right dorsal penile shaft. The absence of symptomatic, erythematous, or erosive mucosal changes typical of classic genital lichen planus favored a pigmentary variant, and a diagnosis of LPP was suspected. He was treated with clotrimazole-betamethasone 1%-0.05% topical cream twice daily in intermittent cycles.

**Figure 1:**
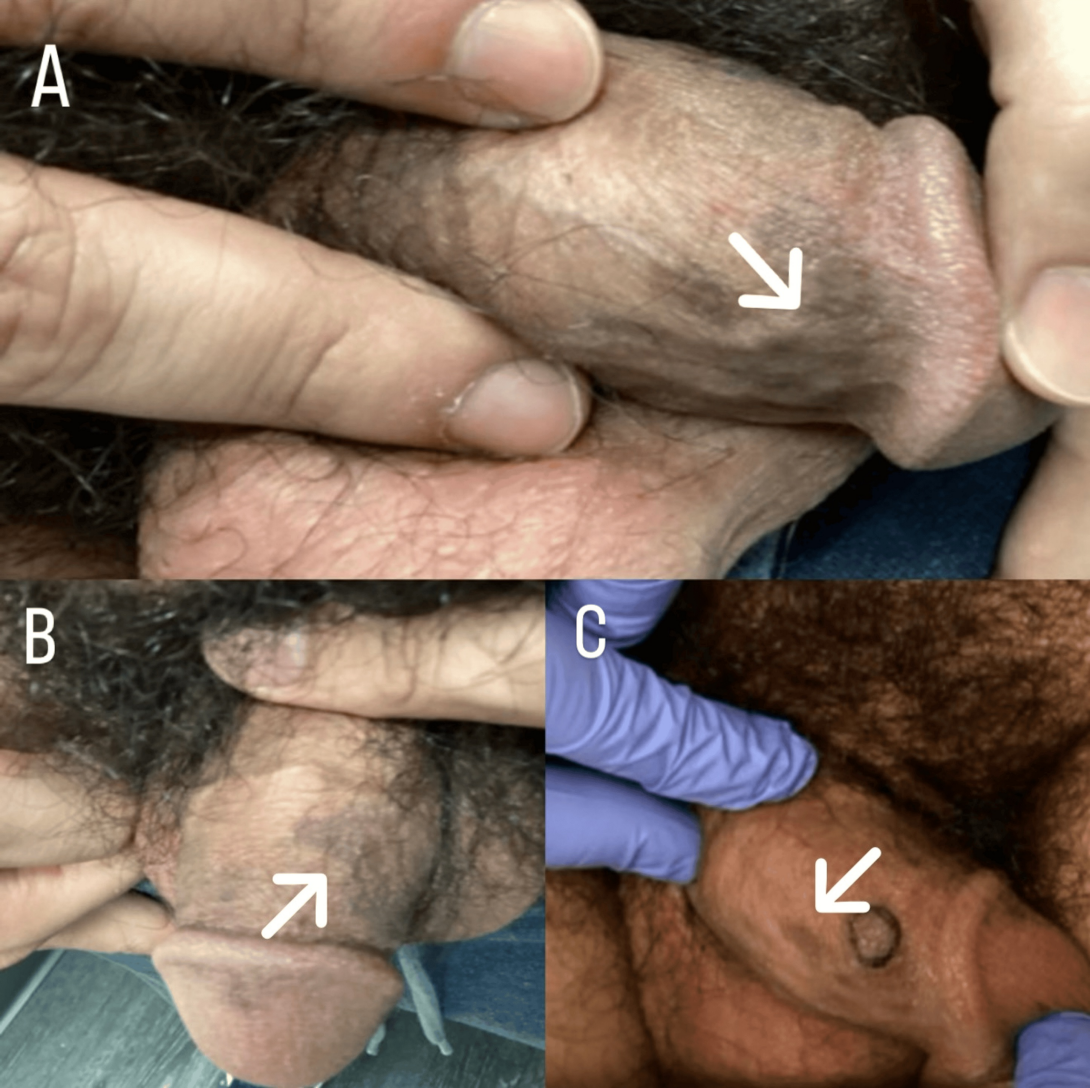
Initial Clinical Presentation of Lichen Planus Pigmentosus on the Glans Penis (A) Close-up clinical photograph demonstrating an ill-defined gray-brown hyperpigmented patch involving the dorsal glans penis (arrow). (B) Additional view highlighting the extent of the hyperpigmented patch on the dorsal glans penis (arrow). (C) Clinical photograph demonstrating a focal hyperpigmented lesion on the dorsal glans penis; the arrow demonstrates the area of interest.

The patient re-presented to an urgent care office in September of 2025 with persistent lesions and was treated with 0.1% topical tacrolimus ointment twice daily. In October of 2025, a physical examination revealed purple, polygonal, lichenoid papules localized to the dorsal corona of the glans penis, as seen in Figures 1A-C. He was prescribed mometasone 0.1% topical ointment to be applied twice daily for up to two weeks, alternating with tacrolimus ointment.

**Figure 2:**
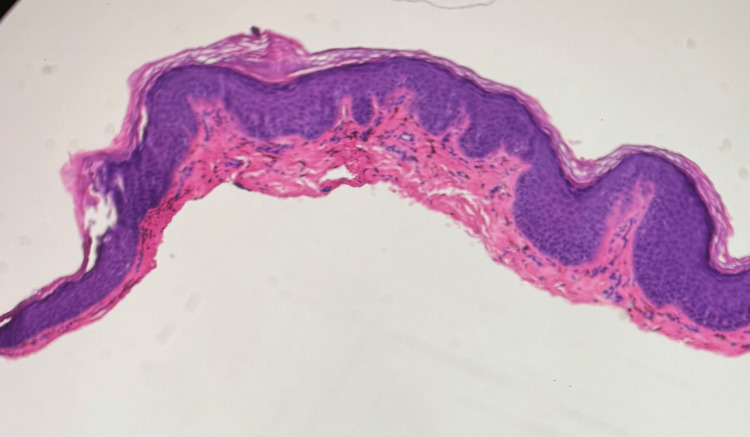
Low-Power Histopathology Demonstrating Lichenoid Inflammation and Wedge-Shaped Hypergranulosis Low-power hematoxylin and eosin (H&E, 4×) section demonstrating a mild lichenoid inflammatory infiltrate along the dermoepidermal junction, with a focal wedge-shaped area of hypergranulosis. Basal vacuolar degeneration (interface change) is present along the dermoepidermal junction, with early pigment incontinence, an important feature of lichenoid dermatitis. The wedge-shaped hypergranulosis is a hallmark of lichenoid inflammation, supporting the diagnosis of lichen planus pigmentosus.

**Figure 3:**
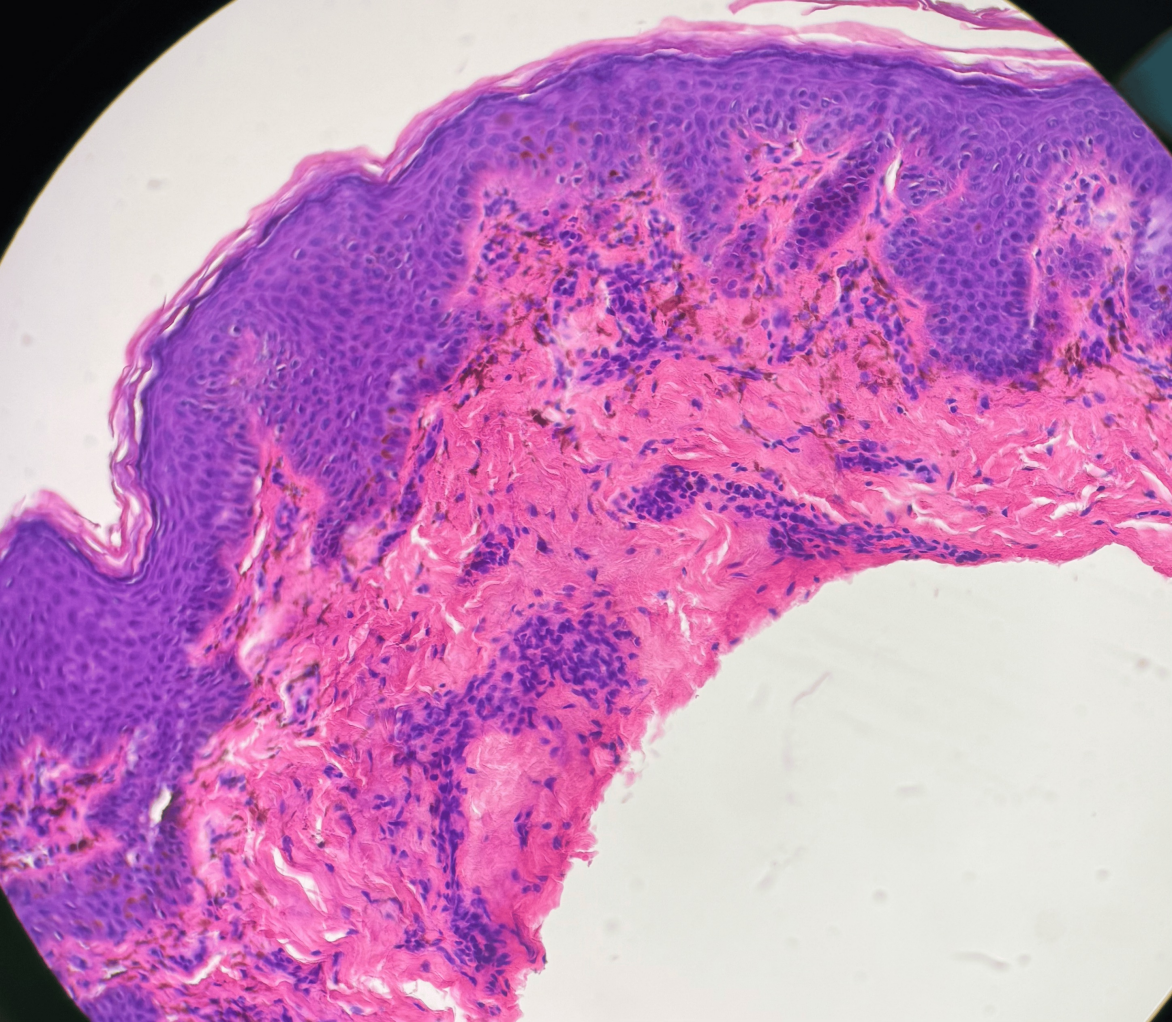
High-Power Histopathology Demonstrating Lichenoid Inflammation With Melanophages and Necrotic Keratinocytes Higher-power hematoxylin and eosin (H&E, 20×) section demonstrating a mild lichenoid inflammatory infiltrate at the dermoepidermal junction with many dermal melanophages (pigment-laden macrophages reflecting pigment incontinence) and scattered necrotic keratinocytes, defining features of lichen planus pigmentosus.

After approximately six weeks without improvement, a shave biopsy was performed to confirm the diagnosis, and histopathologic evaluation was consistent with LPP, demonstrating basal vacuolar degeneration, pigment incontinence, and numerous dermal melanophages. Notably, acanthosis and sawtooth rete ridge elongation, typically seen in classic lichen planus, were absent, supporting the diagnosis of LPP (Figures 2, 3). As seen in Figure 2, a low-power hematoxylin and eosin (H&E) stain demonstrated a mild lichenoid inflammatory infiltrate along the dermoepidermal junction, with a focal wedge-shaped area of hypergranulosis, supporting a lichenoid pattern consistent with LPP. Figure 3 shows a higher-power H&E stain.

**Figure 4:**
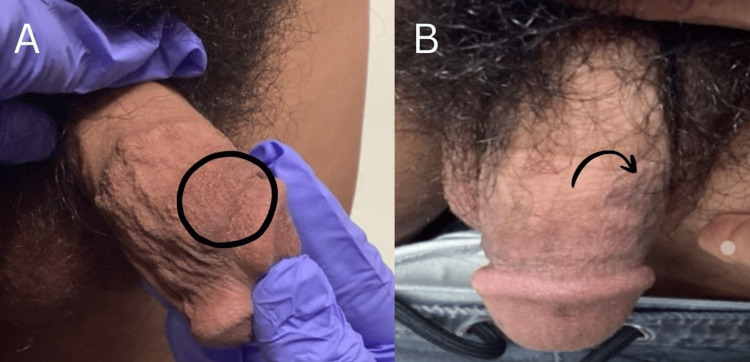
Follow-Up Examination Demonstrating Improvement After Topical Ruxolitinib (A) Follow-up clinical photograph obtained four weeks after initiation of topical ruxolitinib cream applied twice daily, demonstrating overall lightening of the hyperpigmented patch on the dorsal glans penis (circled). (B) Additional follow-up view showing a residual hyperpigmented patch (arrow), with overall interval improvement in pigmentation compared to baseline after four weeks of topical ruxolitinib therapy.

The patient was given topical ruxolitinib samples and instructed to apply twice daily to the affected areas. After four weeks, the patient returned and reported a subtle but noticeable improvement, as seen in Figures 4A, 4B. While complete resolution had not yet occurred, the stabilization of lesions and early fading of hyperpigmentation are promising. A plan was made to continue using ruxolitinib for two to three months and return to the clinic for further evaluation.

## Discussion

LPP is an atypical variant of lichen planus, characterized by dark gray-brown hyperpigmented macules and patches, most commonly on sun-exposed areas of the body such as the face and neck. This disease usually appears in the third and fourth decades of life and is more common in individuals with darker skin, such as those of Indian and Middle Eastern descent. A variant of LPP, called LPP inversus, affects the intertriginous areas such as the axilla. This specific variant of LPP affects individuals with both fair and dark skin and has a worldwide distribution [4]. The scalp, mucosa, and nails are not affected, and isolated involvement of the male genitalia, particularly the glans penis, is rare. Given this uncommon presentation, differential diagnosis includes fixed drug eruption (FDE), erythema dyschromicum perstans (EDP), post-inflammatory hyperpigmentation, and penile melanosis. Genital hyperpigmentation represents a diagnostic challenge because multiple benign inflammatory, drug-related, and pigmentary disorders can present similarly, often encouraging biopsy to reach a differential diagnosis.

EDP is considered the principal differential diagnosis of LPP. It is also characterized by the appearance of asymptomatic ash-colored macules, which later merge and become gray-blue [1]. On histology, LPP more commonly demonstrates a lichenoid dermatitis pattern, consistent with lichen planus, whereas EDP shows prominent pigment incontinence with minimal lichenoid inflammation [1]. Furthermore, EDP classically presents on the trunk and proximal extremities and may also demonstrate an erythematous border early in its course, which is not seen in LPP [1]. In addition, FDE is also important to consider, as the genitalia are often involved, and characteristic recurrence following re-exposure to an offending medication is common. It can manifest as a circulator erythematous patch with or without symptoms of pain and itching and can involve the genitalia depending on the causative drug [5]. These patches can persist for extended periods of time, mimicking chronic pigmentary diagnoses [5]. In contrast, LPP typically develops gradually and is supported by histopathology, as seen in this patient. Therefore, it is important to consider FDE as an alternative diagnosis in front of other penile lesions seen in primary care, such as genital herpes, balanitis, and lichen planus [5].

The initial lesions of LPP are small, brown macules with diffuse borders that later merge to form pigmented gray or brown patches. These patches are typically symmetrical but may be found in a segmental pattern [1]. Histologically, the disease is characterized by vacuolar degeneration of the basal layer with pigment incontinence and keratinocyte apoptosis [1]. The dermis shows a lichenoid infiltrate and incontinence of melanin with scattered dermal melanophages [1]. In contrast to classic lichen planus, which often demonstrates marked epidermal hyperplasia, sawtooth acanthosis, and dense band-like lymphocytic infiltrates, LPP more typically shows a relatively atrophic epidermis with prominent dermal melanophages reflecting chronic pigment incontinence. These histopathologic features were observed in our biopsy specimen and support the diagnosis of LPP over classic inflammatory genital lichen planus.

Although the exact pathogenesis of LPP is unknown, it is thought to result from a T-cell-mediated immune response, in which CD8+ T cells target basal keratinocytes. Subsequent apoptosis causes melanin to enter the dermis, where it is then phagocytosed by macrophages, producing persistent hyperpigmentation. This inflammatory process drives continued pigmentary change and, due to the presence of deep dermal melanophages, may explain why these lesions are resistant to topical corticosteroids and TCIs, particularly in anatomically sensitive regions such as the genitalia. It is also hypothesized that LPP is considered an abortive variant of lichen planus, in which the intensive lichenoid reaction that occurs because of the attack on epidermal keratinocytes by CD8+ T cells occurs quickly before the compensatory increased proliferation of keratinocytes seen in typical lichen planus, which causes the rapid transformation of papules into brown macules in LPP [4].

Management of LPP remains challenging, with many therapies attempted in this disease proving to be ineffective. Current treatment options include calcineurin inhibitors such as tacrolimus, depigmenting agents such as hydroquinone, kojic acid, and hydrocortisone, and systemic treatments such as oral tranexamic acid. Topical steroids are commonly used due to their anti-inflammatory effects but are of questionable efficacy. Whereas they may help broadly suppress inflammation, they do not remove already deposited dermal pigment, and long-term use on genital skin is not ideal. Because tacrolimus is a specific suppressor of T-cell-mediated inflammation, it is considered a reasonable option for LPP; however, it did not prove to be effective in this patient. Furthermore, hydroquinone may address the residual pigmentation but does not address the underlying inflammatory process. In this case, prior therapy with topical corticosteroids and tacrolimus failed to yield meaningful improvement over the course of several months, illustrating the limitation of conventional approaches in LPP.

Recent insights into the immunopathogenesis of lichenoid dermatoses have identified the JAK-STAT signaling pathway as a key mediator of T-cell-driven inflammation. Topical ruxolitinib, a selective JAK1/JAK2 inhibitor, has demonstrated efficacy in treating inflammatory and pigmentary disorders such as vitiligo and atopic dermatitis. The central mechanism behind vitiligo includes the autoimmune destruction of melanocytes, supported by melanocyte-specific autoantibodies and T-cell infiltration in lesional skin [6]. This is also the mechanism behind LPP. Activated CD8+ T cells promote melanocyte apoptosis through IFN-𝛾 and chemokines secreted through the Janus kinase (JAK)/signal transducer and activator of transcription (STAT) signaling pathway [7]. By directly blocking this signaling cascade, JAK/STAT inhibitors prevent STAT protein phosphorylation, suppress CD8+ T-cell activation, and limit inflammation. By halting ongoing damage, macrophages can gradually clear existing melanin, improving hyperpigmentation over time and the appearance of the skin over time. By addressing the root cause of ongoing pigment deposition in addition to inflammation at the surface, JAK-STAT inhibitors such as ruxolitinib show promise.

Only a limited number of case reports have described the use of JAK inhibitors in pigmentary lichenoid disorders. A recent case report demonstrated improvement in a 63-year-old male with recalcitrant LPP involving the bilateral cheeks and anterior neck with topical ruxolitinib [3]. Prior therapy failure included topical steroids, tacrolimus, ketoconazole, hydroquinone, and oral minocycline [3]. Topical ruxolitinib 1.5% cream was applied twice daily, and within three months, a significant reduction in hyperpigmentation was reported, with further reduction of pigmentation by nine months [3]. No side effects were reported. These observations support the notion that JAK-STAT inhibition may attenuate the underlying T-cell-mediated immune process and lead to gradual pigment reduction over time, although the response is neither immediate nor uniformly complete.

Furthermore, in one case report describing a 57-year-old Hispanic female with gray-blue patches on the chest and upper extremities, later confirmed to have EDP arising after COVID-19 vaccination, clinical improvement was demonstrated after one month of topical ruxolitinib 1.5% cream [8]. This finding supports the therapeutic potential of JAK inhibition across the LDP-EDP spectrum and in acquired dermal hyperpigmentation disorders.

In our case, the patient's positive response to topical ruxolitinib highlights the therapeutic potential of targeting JAK-STAT signaling as a novel, steroid-sparing approach, offering the possibility of both stabilizing existing lesions and promoting gradual resolution of hyperpigmented patches. However, the follow-up period in this case was limited to four weeks, which may restrict long-term efficacy and potential recurrence after treatment discontinuation. A longer follow-up will be important to determine the full extent of clinical benefit and to better understand whether JAK inhibition can meaningfully modify disease over time.

## Conclusions

In conclusion, this case illustrates a rare presentation of LPP localized to the glans penis. LPP is thought to result from T-cell-mediated basal keratinocyte damage, leading to apoptosis and melanin deposition in the dermis. The chronic inflammatory nature of this disease makes it resistant to conventional therapies such as topical corticosteroids and TCIs, particularly in sensitive regions like the genitalia. This patient's early clinical improvement with topical ruxolitinib highlights the potential value of targeting the JAK-STAT pathway, which plays an important role responsible for basal keratinocyte apoptosis. By modulating this pathway, ruxolitinib may help reduce ongoing inflammation at the dermoepidermal junction, limiting further pigment incontinence. While longer follow-up and additional studies are needed to better define treatment durability and long-term outcomes, this report emphasizes the importance of considering LPP in the differential diagnosis of persistent genital hyperpigmentation and contributes to emerging evidence supporting JAK inhibition as a potential treatment strategy in pigmentary disorders, especially where conventional therapies are ineffective and risk skin atrophy.
